# “He does not have to wait under a tree”: perceptions of men, women and health care workers on male partner involvement in prevention of mother to child transmission of human immunodeficiency virus services in Malawi

**DOI:** 10.1186/s12913-018-2999-8

**Published:** 2018-03-20

**Authors:** Alinane L. Nyondo-Mipando, Angela F. Chimwaza, Adamson S. Muula

**Affiliations:** 1 School of Public Health and Family Medicine, College of Medicine University of Malawi, Blantyre, Malawi; 2 Malawi Liverpool Wellcome Trust Clinical Research Programme, Blantyre, Malawi; 3 Kamuzu College of Nursing, University of Malawi, Blantyre, Malawi

## Abstract

**Background:**

The perception of male involvement (MI) in maternal child health services is multifaceted and differs among varying programs and populations. In the Prevention of Mother to Child Transmission (PMTCT) context, MI includes men’s attendance at antenatal care (ANC) clinics, undertaking an HIV tests within the ANC and financial and psychological support. Contexualising the definition of MI is fundamental in the development of MI in PMTCT policy and interventions. The objective of this study was to explore the perceptions of men, women and health care workers on male partner involvement in PMTCT services in Malawi.

**Methods:**

A qualitative descriptive study was conducted at South Lunzu Health Centre (SLHC) in Blantyre, Malawi from December 2012 to January 2013. We conducted s Key Informant Interviews (KIIs) with 6 health care workers and moderated four Focus Group Discussions (FGDs) among 18 men and 17 pregnant women attending antenatal care at SLHC. We divided FGDs participants according to sex and age. We digitally recorded all FGDs and KIIs and simultaneously transcribed and translated verbatim into English. We employed thematic analysis to identify codes and themes.

**Results:**

Men and women described MI in PMTCT as either a) Positive participation or b) Negative participation. Positive participation included total involvement of the male partner in PMTCT interventions, reminding the spouse of clinic and treatment schedules, and resource provision. Health care workers described MI as either a) Involvement along the pregnancy continuum or b) Passive Involvement. Participants’ preferred positive involvement of male partners.

**Conclusions:**

There are multiple perceptions of MI in PMTCT with participants preferring positive involvement. There is a need to have a uniform description of MI in PMTCT to optimize development of strategies and interventions that accommodate and optimize MI in PMTCT. A uniform description will be useful in assessing a country’s progress towards achieving MI in PMTCT goals.

## Background

The perception of male involvement (MI) in maternal child health services remains multifaceted and differs among varying aspects of the service. The United Nations Fund for Population Activities (UNFPA) in 1995 defined MI as any support men rendered to women [1]. World Health Organization (WHO) regards MI as the provision of male tailored reproductive health services in addition to services that focus on males and their female partners [2]. The International Conference on Population and Development Programme of Action (ICPD, PoA) emphasized the involvement of men in the quest to improve sexual reproductive health and proposed that countries outline the responsibilities, plans and strategies for involving men [3]. Another contrary form of male involvement promotes male dominance, thus leaving the woman with no decision-making power even regarding her own health issues [4]. In family planning services, adherence to the family planning methods, counseling and uptake of methods such as vasectomies constitute MI [5]. In the PMTCT context, MI comprises a man’s attendance at antenatal care (ANC) clinic with his partner and taking an HIV test within the ANC [6]. Women in a South African study described MI as partner support which included provision of resources including transportation, food and supplemental or replacement infant feeds; reminders of the woman’s PMTCT appointments, which includes encouraging compliance with ART medication and infant HIV testing; emotional support following an HIV positive result; decision making on the mode of infant feeding option [7, 8]; assisting the woman at home with household chores; and interacting with the ANC providers when discussing his partner’s health care issues [9]. Similarly, in Cameroon, women regarded payment of antenatal and other obstetrical bills as male involvement and did not expect anything beyond that from their partners [10].

Notably, the description of MI in PMTCT remains ambiguous and varies with populations and contexts, which imposes challenges in focusing interventions for MI. With the recent highlighted attention towards MI and the claims that the maternal health services have excluded men [11], a clear understanding of MI in PMTCT is vital in developing a male oriented agenda within PMTCT services. Contexualising the description of MI in Malawi is fundamental in the development of policy and interventions for MI in PMTCT. A precise description of MI in PMTCT also serves as a standard benchmark in evaluating the progress towards inclusion of male partners in PMTCT services. The objective of this study was to explore the perceptions of men, women and health care workers on male partner involvement in PMTCT services in Malawi. This study is part of a project on male involvement in PMTCT services. Earlier publications from the project have been about strategies for MI in PMTCT [12], barriers and facilitators to MI in PMTCT [13], relevance of MI in PMTCT [14], effectiveness of an invitation card as a strategy for MI in PMTCT [15] and characteristics and behaviors of men in a PMTCT programme [16]. This paper focuses on descriptions of MI, which was another objective on this project.

## Methods

### Study design and setting

We conducted a qualitative descriptive study at South Lunzu Health Centre (SLHC) in Blantyre, Malawi from December 2012 to January 2013. South Lunzu Health Centre is in the northern part of Blantyre district and serves a semi-urban community. We conducted Focus Group Discussions (FGDs) because they create a social environment where the views of participants on a topic are stimulated not only by one person’s insights but also by the other participants’ views, thereby increasing the quality and richness of the data on the topic [17]. We employed Key Informant Interviews (KII) with health workers because they had a deeper understanding through their experience and expertise on MI in PMTCT. This study was conducted as a component of a formative study to an intervention study on MI in PMTCT in the same setting. We selected the health centre because of its suitability to conduct an intervention study after the formative phase. The semi-urban nature of the area was of interest because it provided data that is applicable both in urban and rural settings [12].

### Sample size

We conducted six Key Informant Interviews (KIIs) with health care workers. Additionally, we conducted a total of four Focus Group Discussions (FGDs) with 17 pregnant women attending antenatal care at SLHC and 18 men from the clinic and its catchment area.

### Selection of study participants

The selection of all study participants has been described prior to this paper [14]. We aimed for maximum variation in the selection of participants to broaden the responses by exploring varying themes across different groups to [18]. We sought consent from all participants prior to their participation in the study. Participants that refused FGD participation cited time constraints as the main reason [14].

We recruited a convenience sample of 18 men for the FGDs with the assistance of health care workers who identified the men based on the criteria that was provided to them. Upon identification of the eligible men, either at the health centre or within the community, the men were asked to report to the health centre at a specific time for a discussion. The inclusion criteria were: 18 years of age and above; identified at the clinic or within the community; fathers with the youngest child below the age of 5, or had a wife who was pregnant; employed, unemployed and/or a business person; willingness to participate in FGDs; and ability to give consent. We limited our criteria above because we assumed such men would have interfaced with PMTCT services and would provide detailed information on the subject. We divided the men into two groups based on age, one with younger men within age range of 18–24 years and the other of older men with age ranging from 25 and above.

We recruited a convenience sample of 17 pregnant women attending antenatal clinic at SLHC to attend FGDs at the health centre. The researcher and research assistant solicited women to participate in the study as they waited for their antenatal appointments. Women who expressed interest to participate in the study were asked to remain for the FGD after their antenatal consultation. Again, we divided the female FGDs into two groups, the younger group which had women with an age range of 18–24 years and an older age group comprising women aged 25+ years. We recruited women that were willing to participate in FGDs, able to consent, had a male partner at home, and were aged 18 years and above. We ensured that we had both primigravidae and multigravidae in the sample.

We recruited a purposive sample of health workers based on their roles and responsibilities in the provision of PMTCT services at the clinic. We recruited informants that attended to pregnant women and their families at different steps of the PMTCT cascade in the course of their duties [18]. The key informants were one medical assistant, two nurse midwife technicians, two HIV Testing and Counseling (HTC) counselors and the PMTCT coordinator for Blantyre district. All interviews were individually scheduled and were conducted in private rooms by the researcher. All informants provided informed consent prior to participation in the study.

### Data collection

Data collection followed pretested interview and discussion guides for the key informants and FGDs respectively. The broad questions that guided the discussion were:

- 1 Would you please describe Male Involvement in Prevention of Mother to Child Transmission of HIV (MI in PMTCT) services in your own terms?
- 2 Would you please describe the current level and type of MI in PMTCT?

We probed further after asking the broad questions in order to gain a more in-depth understanding of the descriptions of MI in PMTCT. Interviews with Key informants lasted for 45–75 min while FGDs lasted for 60–90 min. All interviews and discussions were audio recorded using a digital voice recorder. The Principal Investigator (PI) conducted all key informant interviews in English and Chichewa and facilitated the FGDs in Chichewa with assistance from two protocol-trained research assistants (one male and one female). Data collection continued until saturation was achieved, which was when participants provided no new information. All FGDs and KIIs were simultaneously transcribed and translated verbatim into English. To ascertain validity, we captured participants’ quotes verbatim and conducted the interviews and discussions in the participants’ language. At the end of each interview and discussion, we reiterated the main points for the participants to approve of the main findings. Codes were compared across all transcripts to capture common codes and also highlight the different codes. We followed the Relevance, Appropriateness, Transparency and Soundness of Interpretation Approach (RATS) guidelines in reporting the results of this study [19].

### Data analysis

Transcripts were exported to NVivo 9.0 for management and analysed using thematic analysis. The researcher and an independent researcher performed initial coding; areas of divergence were discussed and normalized. Themes were inductively and deductively realized from the transcripts and research questions, respectively [20]. We employed inductive coding because of the limited literature on the description of MI in PMTCT in Malawi. We also used deductive coding to answer the research questions and allow our results to be compared with findings from other countries according to the available literature. Thematic analysis promoted flexibility during data analysis and allowed for constant code comparison and identification without being restricted to a preexisting theory [21]. Thematic analysis also accommodated the exploration of other findings outside of what is known in the literature, which allowed for a broader understanding of the definition of male involvement in PMTCT [22]. Thematic analysis further assisted in contextualising the description of MI in PMTCT in Malawi [23]. The researcher read the transcripts several times to gain a deeper understanding of the narratives as an act of data immersion. Open coding was performed to the transcripts to designate codes to the data. Similar codes were collated into categories to reduce and present the data in manageable segments which were then arranged under overarching themes. This step also highlighted the codes that were different and was realized after multiple comparisons of the data to ensure that data was appropriately assigned. At the same time, the research questions and literature were used to deductively code the transcripts. In the end, we had both categories that fit in and out of the research question matrix. After developing the overarching themes, the researcher reviewed the themes against the digital recordings to ascertain reliability of the themes and ensure that the themes remain coherent. We were careful to avoid compressing the data too much to preserve the richness and distinctiveness of the findings.

## Results

### Characteristics of study participants

The characteristics of the study participants were reported earlier [12]. In brief, there were eight participants in the younger male age group and 10 participants in the older male age group while there were nine participants in the younger female age group and eight participants in the older age group. All the women and men in the FGDs were married. The median age for the female participants was 18 years and the median gravidity was two pregnancies while the median age for the male participants was 26.5 years and the median age of the youngest child for the male participants was two years old [12].

### Characteristics of key informants

The age range for the key informants was 24–42 years with a median of 30.5 years. Only one out of the six informants was male. Four informants attained tertiary education while the other two attained secondary education.

### Men and women’s perceptions of male involvement in PMTCT services

The focus group participants’ perceptions of male partner involvement in PMTCT were mostly descriptive. The participants indicated that MI in PMTCT of HIV services entails a man taking part in all aspects of antenatal care where PMTCT services are offered. The general expression was that a male partner needs to be involved in order to support the woman and for him to take an HIV test. The following excerpts indicate this finding:

*When we talk about male involvement on HIV issues, it means that in everything that is happening on the wife such as antenatal clinic, HIV testing, a man ought to take part with the wife … a man should take part as follows, accompanying his wife to the hospital (clinic), take an HIV test together, and taking care of the pregnancy and a man should not have (extra marital) relationships. Younger Males (YM) FGDs.*

Some responses from men and women were limited in scope and placed more emphasis on a woman informing her partner about PMTCT services than for a man being involved at the onset of ANC. The following statement illustrates this perception:

*I think it means that when a woman attends to antenatal care and has been asked to have an HIV test, and when she does then she has to inform her husband so that he also knows how he is as well. Younger Females (YF) FGDs*

### Classification of male involvement

In response to the question on the forms of MI, both women and men in FGDs stated that men displayed either positive or negative participation. On the same question, health care workers stated that participation could either be a) Involvement across all stages of Pregnancy or b) Passive participation

### 1. Active participation

Active participation was further discussed as follows: a) Total involvement, b) Reminders, c) Provision, and d) Partial involvement.

- **Total involvement**
  Men and women stated that some men take the leading role in PMTCT services by being available at all steps of the PMTCT process.
*In everything, the man ought to take a leading role if the woman is going to the hospital; if he has time then they have to be together. Additionally, one can create time, one may be busy but when one’s wife is attending to clinic visits a man may leave whatever he was doing to accompany his wife for antenatal care. YM FGD.*
  Some Female FGD participants gave examples of male partner involvement their partners displayed or what they would like their partners to display. The following quotes illustrate this:
*He is with his wife because they have come together and need to listen to everything that will be shared; he does not have to wait under a tree. YM FGD.*
*In my case, he escorts me for my antenatal visits so I see that he is involved … We go in all consultation rooms together. YF FGD*
- **Reminding women of treatment as involvement**
  Women in FGDs voiced that their partners are involved by virtue of their partners reminding them of their dosage times if on medication as well as reminding them of their antenatal appointment dates.
*…sometimes, you (a pregnant woman) may come for antenatal clinic on a day like this one, so you inform him of your antenatal visit and the date of the next appointment … what happens is that as you are nearing the appointment date, he reminds you of the appointment. YF FGD.*
*He is supposed to remind the partner the times when she is supposed to take her medication, if the partner is infected. Older Males (OM) FGD*
- **Provision as participation**
  Only participants from female FGDs mentioned provision of finances and other requirements as a type of involvement men displayed. Some men provide for the needs of their partners and ensure that the woman has transport money for her to get to the health centre.
*Sometimes when you inform him on the antenatal clinic appointment date, he finds money for the wife to buy snacks at the clinic. YF FGD*
- **Partial involvement**
  In some instances, it was noted that there are specific sessions that men attend to during antenatal clinic. It was noted that men are mostly involved in antenatal education and HIV counseling and testing sessions after which they wait for the women to finish up with the rest of the antenatal requirements such as fundal palpations, vaccines and receipt of supplementary medications.
*When men come here, they just attend to the counseling that we are given outside on the things that are needed when a baby is born and also attend to HIV counseling and testing. YF FGD.*
*What they do is as follows: sometimes when the woman comes and she has her weight checked, he goes along, when she goes for counseling he goes along too and both are counseled, and when she gets in the antenatal checking room he waits outside and then they all go home together. Older Females (OF) FGD*

### 2. Negative participation

It also emerged from both male and female FGDs that in some cases a man participates negatively by refusing to take an HIV test and also by disapproving of his wife’s decision to take an HIV test.

*…we can say that he has participated by refusing (negative participation) because there are two sides, accepting or refusing so it means if he refuses then he has participated negatively. YM FGD*

### Health care workers’ perceptions of male partner involvement

- **Involvement across all stages of pregnancy**
  The health care workers’ perceptions of male involvement focused on all the stages of pregnancy. They stated that a male partner is expected to participate from pre-conception to post delivery.
  *MI in PMTCT is like involving men in PMTCT of HIV in all areas … before the woman is pregnant, during pregnancy and after (delivery). KII respondent*.
  Health care workers mentioned that male partners’ total involvement has resulted in a cultural change whereby the support rendered by a woman’s mother or another elderly woman is offered by the male partner instead.
*He always follows whatever you are doing, during examination, he is there to know what you are doing … even sometimes they encourage the woman whenever at home … nowadays we can see even grand mums and even mothers, they do not come all the way from the village, staying with their pregnant daughters, we see these men, carrying the basket following the wife at the hospital because of that. KII Respondent*
- **Passive participation**
  Health care workers stated that in some instances men passively participate in PMTCT of HIV services. They are reduced to a silent participant who mainly watches, necessitating a provider’s initiative to ensure that men are actively involved. The quotes below illustrate this.

*Most of the men that I have met are always quiet. But if we involve them by asking those questions, that is when they open up and ask questions but most of them are just quiet, they will just be listening, if you do not ask questions, if you do not involve them, they will just be quiet. KII Respondent.*

*…when they are here they stand far off and the woman would be attended to, only when one realizes that the man is to consult together with the partner for fast attendance. KII Respondent.*

Health care workers also reiterated that in some cases they take the initiative to invite the male partner once they have noted some passivity on the man.

*Most of the time they stay aside, so if you ask her (the wife) she will say “we are partners” and we ask her to just call him so that he should be in the conversation as well. KII Respondent*

## Discussion

The main findings of our study showed perceptions of MI in PMTCT varied among different participants. Men and women’s perceptions emphasized the support a man renders to a woman while health care workers narrowed their perceptions to the specific activities that a man is involved in. Our study showed that there are various forms of MI, such as positive, negative and passive involvement across all stages of pregnancy.

Our finding that MI entails a range of activities that men do or ought to do remains consistent with previous research in South Africa [7, 8]. The similar findings focused on provision of resources and reminders of antenatal appointments. The difference was that our study further described male involvement as a man being fully involved in all aspects pertaining to ANC. An aspect that was not highlighted in our study was the emotional support rendered to a woman by her partner when a woman learns of her HIV status which Maman et al. [7] previously reported. This could be partially explained by the design of our study; HIV infection was not a criterion as was the case in the other study. As such, we did not ask direct questions about the support HIV infected women received after learning their status. In addition, our study provided a range of views of descriptions of MI compared to a Cameroonian study where women only referred to payment of antenatal fees as MI [10].

Our findings on the various types of involvement remain congruent with the findings in a South African study where men were classified into active, reluctant (but could be motivated) and uninterested groups in PMTCT services [24]. The active involvement of men in PMTCT, as expressed in this study, builds on the recommendation in a South African study where emphasis was placed on male participation transcending beyond mere presence at the antenatal clinic to include men learning and gaining information to promote the health of their spouse and baby [25]. We recommend developing guidelines that outline what MI entails to ease with assessing the form of participation that men display.

The partial participation expressed in this study is consistent with findings by Ladur et al. [24], except that in the latter study men were only allowed to participate in the labour room while in our study their participation was limited to antenatal education and HIV counseling and testing. Our finding that HIV counseling and testing is the only services that that directly benefits men is consistent with a review by Sherr et al. [26]. We propose other screening services for men’s reproductive health that can be incorporated in the service to avoid reducing men to silent partners. Furthermore, a clear definition of the role of men in PMTCT needs to be outlined to assist with program implementation [24]. This finding and recommendation underscores suggestions from previous studies that support the incorporation of men in reproductive programmes from conceptualization of the project other than limiting their involvement to inactive partners that are “escorting” their spouses [27]. Additionally, reproductive programs should have other services that include men’s health as a goal [26]. The partial form of involvement offers men limited information resulting in an incomplete understanding of the whole ANC/PMTCT service. For instance, some aspects about antiretroviral are discussed during consultation with a midwife during abdominal examination.

The passive form of involvement expressed by participants in this study was also reported by Maman et al. [7] where women reported a lack of involvement from their male partners in PMTCT. Passive involvement could be perpetuated by cultural norms that dictate that an ANC is a woman’s space [28–31] with men being left with the role of financing the home [8, 31, 32]. Furthermore, it is common practice in African settings for other female relations to support a pregnant or newly delivered woman [32]. Additionally, the organization of health services also contributes to male passive involvement by implementing rules that prohibit involvement of men in some sections within maternal services [8, 29]. The absence of a policy defining and supporting MI in PMTCT encourages passive participation and makes it a challenge for an employee to be released from work for that exercise.

Male negative participation illustrated by preventing their partners accessing PMTCT services, as stated in this study, remains congruent with earlier findings where men were reluctant to be involved owing to stigma [24]. Negative participation has the potential to escalate intimate partner violence [33, 34]. In addition, it contributes to a woman delaying HIV testing and antiretroviral initiation until her partner has consented [35–37], which creates a missed opportunity for early treatment or may result in non-adherence to antiretroviral [38]. In the Malawian context, this form of participation was previously explained by earlier results from this project, that men refrained from any form of involvement or their spouse’s involvement to avert learning their own HIV status directly or indirectly [13]. Negative participation thrives on the cultural underpinnings that confer powers to men as highlighted in Sub Saharan Africa, where men greatly influence women’s health seeking behaviors, [39, 40] including decisions regarding maternity [41], irrespective of the prevailing belief that maternity issues fall under a woman’s domain. The negative participation is worsened by the diminishing decision-making power that some women have even on matters relating to their own health [42].

Although our study participants preferred total involvement from a male partner, as previously reported by Ladur et al. in South Africa [24], achieving this level of involvement has its logistical and economic challenges since most men are the breadwinners within their homes, thereby imposing a challenge for a man to attend to PMTCT services in the current settings [31]. Achieving total male partner involvement would require normalizing their participation to a level where men and society would regard it as an expected activity [27]. Normalization of MI in PMTCT may be achieved by overcoming identified barriers and capitalizing on enabling factors [13, 30] with an intention of challenging the prevailing gender norms [27].

### Strengths and limitations

Although useful insights can be drawn from the findings of this study, the results may not be generalized to other settings since the sampling technique employed does not allow for that. We explored perceptions which yielded different findings compared to observing practices. The findings in our study echo those of previous researchers. Our study was conducted in a semi-urban setting which is different from other settings where previous studies were conducted. This study is among the limited studies that have reported on the perceptions of MI in PMTCT from multiple stakeholders’ offering a comprehensive view which may provide insights when defining MI in PMTCT.

## Conclusions

There are multiple perceptions of MI in PMTCT with participants preferring positive involvement from male partners. We recommend development of a comprehensive but uniform description of MI in PMTCT to optimize development of strategies and interventions that accommodate and increase MI in PMTCT. A uniform description will be useful in assessing a countries’ progress towards achieving MI in PMTCT goals. Policy makers should lay out a clear definition of the core values for the service and ought to deliberately state the male tailored health services within the program.

## Abbreviations

ANC: Antenatal care
ART: Antiretrovirals
CARTA: Consortium for Advanced Research Training in Africa
COMREC: College of Medicine Research and Ethics Committee
DelPHE: Development Partnerships in Higher Education
DfID: Department for International Development
FGDs: Focus Group Discussions
HIV: Human Immunodeficiency Virus
HRCSI: Health Research Capacity Strengthening Initiative
ICPD: International Conference on Population and Development Programme of Action
KIIs: Key Informant Interviews
MI: Male Involvement
OF: Older Females
OM: Older Males
PI: Principal Investigator
PMTCT: Prevention of Mother of Child Transmission
RATS: Relevance Appropriateness Transparence and Soundness
SLHC: South Lunzu Health Centre
UNFPA: United Nations Fund for Population Activities
WHO: World Health Organisation
YF: Young Female
YM: Young Male
